# Supplementary material for: Identification of photoperiod-induced specific miRNAs in the adrenal glands of Sunite sheep (Ovis aries)
Source: Front Vet Sci. 2022 Jul 22;9:888207. doi: 10.3389/fvets.2022.888207 (PMC9354845; doi:10.3389/fvets.2022.888207)
Supplement: Supplementary file 1 [file Data_Sheet_1.ZIP › Supplementary file/miRDeep2 file.zip!/miRDeep2 file/Know_Structure/SPLP42A3/oar-mir-370.pdf]

miRBase precursor : oar-mir-370
Total read count : 14508
oar-miR-370-5p read count : 34
oar-miR-370-3p read count : 14460
remaining reads : 14

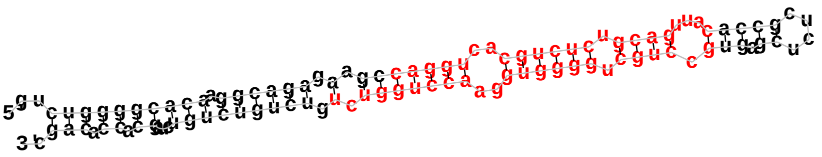

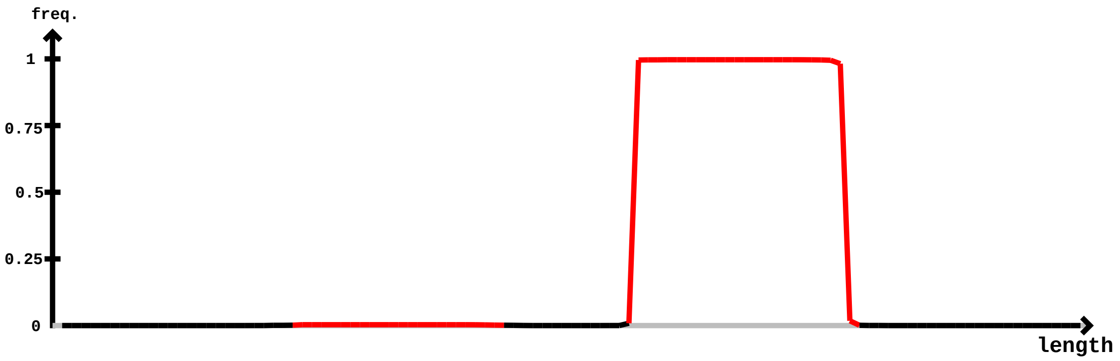


```
                                                                                                   oar-miR-370-3p
                                             oar-miR-370-5p
5'- gucuggggcacaaggcagagaagccaggucacgucucugcaguuacaccgcucucgagugccugcugggguggaaccuggucugucugucugucuagcaccacagc   -3'    exp
    ..((((((((((.((((((.(.(((((((..((((((.((((...(((((....)).))).)))).))))))..))))))).).)))))))))...)).)).))).     reads      mm        sample
    ...................gaagccaggucacgucucug...................................................................     1          0         seq
    .....................agccaggucacgucucugcag................................................................     2          0         seq
    .....................agccaggucacgucucugcagu...............................................................     8          0         seq
    .....................agccaggucacgucucugcaguu..............................................................     2          0         seq
    .......................ccaggucacgucucugcaguu..............................................................     5          0         seq
    .......................ccaggucacgucucugcaguua.............................................................     1          0         seq
    .......................ccaggucacgucucugcaguuaca...........................................................     2          0         seq
    ........................caggucacgucucugcaguu..............................................................     3          0         seq
    ........................caggucacgucucugcaguua.............................................................     6          0         seq
    ........................caggucacgucucugcaguuac............................................................     6          0         seq
    ........................caggucacgucucugcaguuaca...........................................................     7          0         seq
    ........................caggucacgucucugcaguuacac..........................................................     4          0         seq
    ........................................................agugccugcugggguggaaccuggu.........................     1          0         seq
    ..........................................................ugccugcugggguggaac..............................     1          0         seq
    ..........................................................ugccugcugggguggaaccugg..........................     4          0         seq
    ..........................................................ugccugcugggguggaaccuggu.........................     118        0         seq
    ..........................................................ugccugcugggguggaaccugguc........................     3          0         seq
    ...........................................................gccugcugggguggaacc.............................     3          0         seq
    ...........................................................gccugcugggguggaacU.............................     1          1         seq
    ...........................................................gccugcugggguggaaccu............................     6          0         seq
    ...........................................................gccugcugggguggaaccug...........................     18         0         seq
    ...........................................................gccugcugggguggaaccugg..........................     182        0         seq
    ...........................................................gccugcugggguggaaccuggu.........................     13864      0         seq
    ...........................................................gccugcugggguggaaccugguc........................     236        0         seq
    ...........................................................gccugcugggguggaaccuggucu.......................     8          0         seq
    ............................................................ccugcugggguggaaccuggu.........................     4          0         seq
    ............................................................ccugcugggguggaaccugguc........................     6          0         seq
    .............................................................cugcugggguggaaccuggucu.......................     1          0         seq
    ..............................................................ugcugggguggaaccuggucugu.....................     5          0         seq
```
